# A Hybrid Approach for Noise Reduction in Acoustic Signal of Machining Process Using Neural Networks and ARMA Model

**DOI:** 10.3390/s21238023

**Published:** 2021-12-01

**Authors:** Tayyab Zafar, Khurram Kamal, Senthan Mathavan, Ghulam Hussain, Mohammed Alkahtani, Fahad M. Alqahtani, Mohamed K. Aboudaif

**Affiliations:** 1 College of Electrical & Mechanical Engineering, National University of Sciences and Technology (NUST), Islamabad 44000, Pakistan; tayyab.zafar@ceme.nust.edu.pk (T.Z.); khurram.kamal@pnec.nust.edu.pk (K.K.); 2 Department of Civil and Structural Engineering, Nottingham Trent University, Burton Street, Nottingham NG1 4BU, UK; s.mathavan@ieee.org; 3 Faculty of Mechanical Engineering, GIK Institute of Engineering Sciences and Technology, Topi 44000, Pakistan; ghulam.hussain@giki.edu.pk; 4 Department of Industrial Engineering, College of Engineering, King Saud University, P.O. Box 800, Riyadh 11421, Saudi Arabia; afahad@ksu.edu.sa (F.M.A.); maboudaif@ksu.edu.sa (M.K.A.)

**Keywords:** noise reduction, neural network, airborne acoustic emission, ARMA

## Abstract

Intelligent machining has become an important part of manufacturing systems because of the increased demand for productivity. Tool condition monitoring is an integral part of these systems. Airborne acoustic emission from the machining process is a vital indicator of tool health, however, it is highly affected by background noise. Reducing the background noise helps in developing a low-cost system. In this research work, a feedforward neural network is used as an adaptive filter to reduce the background noise. Acoustic signals from four different machines in the background are acquired and are introduced to a machining signal at different speeds and feed-rates at a constant depth of cut. These four machines are a three-axis milling machine, a four-axis mini-milling machine, a variable speed DC motor, and a grinding machine. The backpropagation neural network shows an accuracy of 75.82% in classifying the background noise. To reconstruct the filtered signal, a novel autoregressive moving average (ARMA)-based algorithm is proposed. An average increase of 71.3% in signal-to-noise ratio (SNR) is found before and after signal reconstruction. The proposed technique shows promising results for signal reconstruction for the machining process.

## 1. Introduction

With the increase in demand for unmanned manufacturing processes, intelligent machining has become indispensable to avoid breakdown maintenance [1]. Scheduled maintenance is considered as an effective tool to increase a manufacturing system productivity [2]. Tool breakage is a major cause of breakdown maintenance in an industrial setup which not only increases the manufacturing cost but also wastes time [3]. Moreover, machine tool quality degrades with time, which affects the product quality. Hence, predicting the tool’s health in time is of critical importance to enhance the product quality as well as to save cost and time. To monitor the tool health, various methods are introduced in the literature. These methods can be divided into two groups: direct methods and indirect methods [4]. Direct methods are based on the physical geometry of a tool that directly indicates the tool condition. These methods involve image processing, electrical resistance, and radioactive based techniques to evaluate tool health. These methods are generally accurate in predicting tool health; however, they require stopping the machine which not only interrupts production, but may also increase the production cost. To avoid these issues, indirect methods are introduced. Indirect methods are based on the signals that may generate from the machining process. These methods involve various signals—such as cutting force, temperature, vibration from the tool and tool holder, acoustic emission, etc.—from the machining process that can be used to observe the tool condition [5].

Ghosh et al. [6] estimated average flank wear of a main cutting edge of tool for a milling process using sensor fusion. They used a combination of seven sensors including cutting force sensor, tool, and work-piece vibration measurement sensor, spindle current and voltage sensors, sound pressure level sensor, and acoustic emission (AE) sensor. Among these signals, AE based methods are preferred over others due to their higher frequency range [7]. Acoustic emission can be divided into two categories, structure-borne acoustic emission and air-borne acoustic emission [8]. Structure-borne acoustic emission is the vibrations that are transferred from the tool to the tool-holder. For a metal cutting process, the characteristic frequency of the emission lies with-in the range of 500 kHz to 1 MHz (ultrasonic range). Acoustic emission-based methods for the machining process are reviewed in [9]. The structure-borne acoustic emission being having an ultrasonic frequency range is an environmental noise free technique; however, it requires high computational processing power. Air-borne acoustic emission is the vibrations that are transferred from cutting edge to the surrounding. The air-borne acoustic emission lies within audible range—i.e., from 0 Hz to 20 kHz. Different methods using airborne acoustic emission to monitor the tool health have been proposed by researchers. The relationship between air-borne acoustic emission and tool vibrations is determined by [10]. They found that the vibrations from the tool holder, along with the cutting insert, are a major source of sound generation. Delio et al. [11] proposed a method to detect chatter using airborne acoustic emission. Their results show that the performance of the airborne acoustic emission-based chatter detection approach is comparable with other methods based on displacement probes, accelerometers, and dynamometers. Sari et al. [12] studied the effect of various micro-piercing parameters to the power spectrum of the airborne acoustic emission. It is observed that cutting processes with different materials, sheet thicknesses, and clearances present a response in the range of 3–4 kHz For a honing process, Corral et al. [13] developed an airborne acoustic emission based method to monitor the surface roughness, material removal rate and tool wear. Kopac and Sali [14] developed a tool monitoring system using airborne acoustic emission. They conducted several experiments with different cutting speeds, feed-rates, and different amounts of wear. The depth of cut was taken as a constant in the experiments. They concluded that the frequency range depends upon both cutting speed and feed rate; however, the effects of cutting speed are lesser on the range than the feed-rate. A two-stage technique is developed by Aliustaoglu et al. [15] to monitor the tool condition using airborne acoustic emission. Several experiments were conducted on a four-axis CNC machining center. Their results show that the proposed method can identify the various tool wear conditions. Another airborne acoustic signal-based system is developed by Salgado and Alonso [16] to monitor the tool wear in turning processes. They acquired the feed motor current and acoustic signals. They analyzed the acquired signals using singular spectrum analysis. Support vector machine (SVM) was then utilized as a learning model to predict the tool wear. Kothuru et al. [17] studied the use of airborne acoustic signals to predict the cutting tool wear and failure during the end milling operation. The signals were acquired from different positions and analyzed in the frequency domain to extract features that correlate the cutting phenomenon. Their method utilized SVM as a decision-making algorithm. Another approach to predict the remaining life of a tool using air-borne acoustic emission can be found in [18].

One of the major challenges in the implementation of the airborne acoustic emission in industrial applications is environmental noise from the machine surroundings [19]. Ruiz-Carcel et al. [20] proposed the spectral kurtosis as a tool to increase the signal-to-noise ratio of acoustic emission for roll bearing acoustic emission. The major advantage of the technique is the automatic selection of band-pass frequency to increase the SNR. Zhu et al. [21] developed an algorithm to de-noise a signal where the signal is affected by non-Gaussian noise. Blind source separation was used for this purpose. For a micro-milling process, Lu and Wen [22] attempted to reduce the background noise effect on the acquired signal using hidden Markov model (HMM). Seemuang et al. [23] studied the relationship between spindle noise and tool wear for a cutting process. It is found that there is no relationship between frequency of spindle noise and tool wear. However, changing the various parameters such as cutting speed and feed rate have significant effect of spindle noise magnitude. A tool breakage detection technique using empirical mode decomposition and independent component analysis is developed by Shi et al. [24]. The technique was able to detect tool breakage by de-noising signals during face milling. Li et al. [25] proposed a technique to de-noise the acoustic emission of a milling operation. The features were extracted in time-frequency domain and an adaptive kernel principal component analysis was performed to de-noise the acquired signals. In the time-frequency based analysis techniques, synchrosqueezing-transforms-based techniques are developed [26]. Tary and Herrera [27] proposed continuous wavelet and synchrosqueezing-transform-based method to denoise the signals. Sony and Sadhu [28] developed a hybrid time–frequency based method combing multivariate empirical mode decomposition and synchrosqueezing transform to identify the time-varying behavior of the signal. Generally, the techniques to de-noise the acoustic emission are developed based on the frequency analysis of the acquired signal. However, a frequency-based filtering technique cannot be applied as there is a chance of losing frequency of interest within the range specified for airborne acoustic emission. For a machine shop, there exists a specific type of noise that is emitted from the background machines present in that workshop and, hence, a neural-networks-based filtering approach is best suitable to identify such type of noise through adaptive filtering. A neural-network-based noise reduction technique for a machining process was proposed by Zafar et al. [29]. The neural network performance was compared with the unsupervised and clustering approaches.

The aim of this paper is to develop a neural network and ARMA-based algorithm to reduce background noise for a machining signal. A machining signal can be affected by a variety of background noise for example, such as sounds from parallel running machines, operator voice or sounds in background; however, in this research, noise generated by parallel running machines on the shop floor are only considered. Acoustic signals from four different background machines are acquired and are introduced to the machining signal as sources of noise. Feedforward neural network is used as an adaptive filter to reduce the background noise. The proposed method adapts itself according to environmental conditions and extracts the machining signal from background noise effectively. The filtered signal is reconstructed through an ARMA-based algorithm which is a novel approach. The contributions of the paper can be summarized as follows: (1) the proposed method utilizes a novel neural network and ARMA-based algorithm to reduce the background noise. (2) An ARMA-based algorithm is developed find the ARMA parameters automatically which is then utilized to reconstruct the filtered signal. (3) The proposed approach extracts the statistical features of the acquired signals in the time domain. (4) The developed approach is benchmarked for several experiments for different levels of noise.

The rest of the paper is organized as follows: Background theory of the developed algorithm is provided in Section 2. Section 3 discusses proposed method to reduce the background noise and the signal reconstruction. Section 4 describes the detailed experimental setup to acquire acoustic signals. Results and discussion for the acquired signals filtration and reconstruction are provided in Section 5. Section 6 discusses various challenges to the research implementation in an industrial environment and the conclusions.

## 2. Background Theory

**Figure 1:**
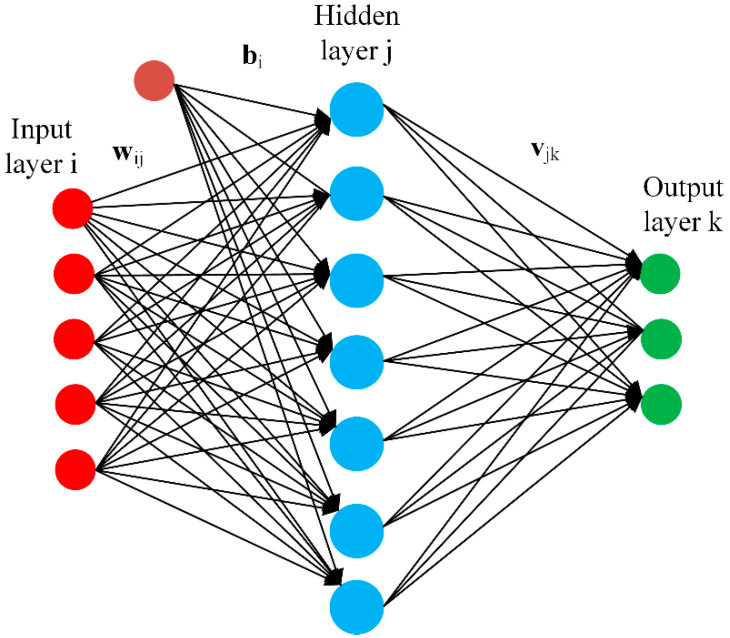
Feedforward neural network structure.

Typical architecture of a neural network is shown in Figure 1. A neural network is a network of interconnected neurons which are organized in three layers. These layers are typically called input layer i, hidden layer j, and output layer k. The neural network attempts to build a mathematical model between a set of feature vectors $X_{ii}\;\epsilon \;{\mathbb{R}}_{n}$ and their corresponding response $Y_{ii}\;\epsilon \;{\mathbb{R}}_{p}$ and hence the learning data can be presented as $\varrho ={\left[\left(X_{k},Y_{k}\right)\right]}_{k=1}^{E}$. Typically, set of feature vector is taken as input to the neural network and their response is taken as output of the network. Hence, the number of neurons in input and output layers are equal to feature vector dimensions and the response respectively. However, the number of neurons in the hidden layer can be variable and can be selected based on the experience.

The neural network is designed to model a relationship between the input feature vectors $X_{ii}$ and their corresponding response $Y_{ii}$. A set of weight or gain factors $w_{ij}$ as well as a bias b is assigned to each connection between the input and hidden layers. Mathematically, the hidden layer neurons response γ are given as

(1) $$\gamma =\sum^{\;}w_{ij}X_{ii}+b$$

An activation function S(γ) is used to identify the state of input layer neurons. For this work, a typical sigmoid function is used which can be given as

(2) $$S\left(\gamma \right)=\frac{1}{1+e^{-y}}$$

The sigmoid function output lies within the range of [0, 1]. The sigmoid function response is again multiplied with a new set of weights $v_{jk}$. The same procedure is repeated between the hidden layer and the output layer. The weight values are updated in iteratively to minimize the error between the desired response $\;Y_{ii}$ and the predicted response.

(3) $$W_{kk}=W_{kk-1}+\Delta W_{kk}$$

where kk is the total number of iterations to reach desired mean squared error and $\Delta W_{kk}$ is the change in weight. The mean square error is used to minimize the error, which is given as

(4) $$MSE=\frac{1}{N}\overset{N}{\underset{ii=1}{\sum }}{\left({\hat{Y}}_{ii}-Y_{ii}\;\right)}^{2}$$

where N is the total number of training samples and ${\hat{Y}}_{ii}$ is the predicted response. Various algorithms are used in the literature to update weights. These algorithms include scaled conjugate gradient method [30], Bayesian interpolation [31], and Levenberg–Marquardt (LM) algorithm [32], etc. In this research, an LM based backpropagation algorithm is employed because of its fast convergence. The algorithm adapts the weights of the neural network using the weight update rule

(5) $$\Delta W_{kk}=-\mu {\left[H\left(W_{kk-1}\right)\right]}^{-1}\nabla E\left(W_{kk-1}\right)$$

where μ is the learning rate, H is the Hessian matrix, and ∇E is the gradient of the network error function. The neural network is used to filter the machining signal.

To reconstruct the filtered signal, a novel ARMA-based algorithm is developed. ARMA is a time series model that has been used in other fields of research to predict the forecast of different systems such as sunspot data, rain prediction systems, prediction of dam inflow of a dam reservoir, etc.; however, it has not been used to reconstruct the signal.

For a typical timeseries such as the predicted neural network response $\;y_{1},y_{2},\ldots ,\;y_{t}$, the ARMA model is a combination of auto-regressive model of order (*p*)

(6) $$y_{t+1}=y_{t}+\varphi_{1}y_{t-1}+\cdots +\varphi_{p}y_{t-p}+𝛿$$

and moving average model of order (*q*)

(7) $$y_{t+1}=\varepsilon_{t}+\theta_{1}\varepsilon_{t-1}+\cdots +\theta_{q}\varepsilon_{t-q}$$

where $\emptyset_{i},\theta_{i}$ are the model coefficients, $y_{t}$ is the previous sample value and $\varepsilon_{t}$ is the white noise error. In general, the autoregressive model determines that the current value of the system depends on how many previous terms whereas, the MA models are ‘averages’ of the past and present noise terms.

AR and MA model can be combined to form ARMA which can be mathematically defined as

(8) $$y_{t}=y_{t}+\emptyset_{1}y_{t-1}+\cdots +\emptyset_{p}y_{t-p}+\varepsilon_{t}+\theta_{1}\varepsilon_{t-1}+\cdots +\theta_{q}\varepsilon_{t-q}$$

To estimate the ARMA model, Box and Jenkins method [33] is used here. There are two basic steps of the ARMA model estimation, namely, identification of ARMA order and estimation of ARMA coefficients.

### 2.1. Identification of ARMA Model Order

To identify the correct order of the ARMA model automatically two tests are used; namely autocorrelation function ACF test and partial autocorrelation function PACF test. Autocorrelation function test shows how correlated the observations are, that is h lags apart, and is used to identify the order of the MA model. Mathematically, ACF for a set of measurements $y_{1},y_{2},\ldots y_{T}$ can be defined as

(9) $$r_{h}=\frac{C_{y}\left(h\right)}{C_{y}\left(0\right)}$$

where $C_{y}\left(h\right)$ is given by

(10) $$C_{y}\left(h\right)=\frac{1}{T}\overset{T-h}{\underset{g=1}{\sum }}\left(y_{g}-\bar{y}\right)(y_{g+h}-\bar{y})$$

The number of peaks outside the predefined upper and lower bounds determines the order of the MA model. The PACF is interpreted as a regression of the series against its past lags and is used to determine the order of the AR model. Mathematically, PACF can be calculated as

(11) $$\chi_{kk}=\frac{\left|\begin{array}{cccc} 1 & r_{1} & \cdots & r_{1}\\ r_{1} & 1 & \cdots & r_{2}\\ \vdots & \vdots & \ddots & \vdots \\ r_{k-1} & r_{k-2} & \cdots & r_{k} \end{array}\right|}{\left|\begin{array}{cccc} 1 & r_{1} & \cdots & r_{k-1}\\ r_{1} & 1 & \vdots & r_{k-2}\\ \vdots & \vdots & \ddots & \vdots \\ r_{k-1} & r_{k-2} & \cdots & 1 \end{array}\right|}$$

Similarly, PACF indicates the order of the AR model. AR model will be of $M^{th}$ order if M peaks are outside the 95% confidence level. One of the pre-requisite conditions of the time series model is that the series must be stationary [34]. To make the series stationary, $M^{th}$ order difference can be taken. Generally, the value of M is set to be 1 or 2; however, care must be taken in selecting the value of M to avoid under or over differencing of the series. An under differenced series may behave as non-stationary series while an over differenced series may behave as a stationary series; however, the estimation of coefficients would be difficult in this case. There are two tests to check the proper order of the difference for the series—ACF test and variance test. ACF plot can be used to test the under differencing of the series. If ACF of the series dies out quickly, then it means that the series is stationary, otherwise, the series is non-stationary and series must be differenced. To determine the over differencing of the series, variance test can be used—i.e., the variance of a stationary series is minimum. It means that variance of the different order differenced series can be recorded and the minimum value of variance will determine the proper order of difference that is needed to be taken to make the series stationary.

### 2.2. Estimation of ARMA Coefficients

The second step of ARMA model estimation is to determine the value of its coefficients. This is done by the maximum likelihood method (MLE). The method selects the values as estimators of a set of parameters that maximize $L\left(q_{1},q_{2},q_{3},\ldots ,q_{T}\right)=f\left(y_{1},y_{2},y_{3}\ldots ,\;y_{T};\;q_{1},q_{2},q_{3},\ldots ,q_{T}\right)$ where $f\left(y_{1},y_{2},y_{3},\ldots ,\;y_{T};q_{1},q_{2},q_{3},\ldots ,q_{T}\right)$ is the joint density function of the observations $y_{1},y_{2},y_{3},\ldots ,\;y_{T}$. $L\left(q_{1},q_{2},q_{3},\ldots ,q_{T}\right)$ is called the likelihood function. Finding the values $q_{1},q_{2},q_{3},\ldots ,q_{T}$ to maximize $L\left(q_{1},q_{2},q_{3},\ldots ,q_{T}\right)$ is equivalent to finding the values to maximize $l\left(q_{1},q_{2},q_{3},\ldots ,q_{T}\right)=\ln\left(L\left(q_{1},q_{2},q_{3},\ldots ,q_{T}\right)\right)$ which is called the log-likelihood function [34].

To estimate the p+q+2 parameters $\varphi_{1},\varphi_{2},\varphi_{3},\ldots ,\varphi_{p};\theta_{1},\theta_{2},\theta_{3},\ldots ,\theta_{q};\delta ;\sigma^{2}$ of the ARMA model using MLE, joint density function of the observations $y_{1},y_{2},y_{3},\ldots ,\;y_{t}$ needs to be found—i.e., $f\left(\varphi_{1},\varphi_{2},\varphi_{3},\ldots ,\varphi_{p};\theta_{1},\theta_{2},\theta_{3},\ldots ,\theta_{q};\delta ;\sigma^{2}\right)=f(y|\varphi ,\theta ,\delta ,\sigma^{2})$. It is difficult to determine the exact density function of $y_{1},y_{2},y_{3},\ldots ,\;y_{t}$ from this information; however, if it is assumed that p starting values on the AR model $s^{*}=s_{1-p},s_{2-p},s_{3-p},\ldots ,\;s_{0}$ and q starting values on the MA model $u^{*}=u_{1-p},u_{2-p},u_{3-p},\ldots ,\;u_{0}$ have been observed then the conditional distribution of given $y_{1},y_{2},y_{3},\ldots ,\;y_{N}$, $s^{*}=s_{1-p},s_{2-p},s_{3-p},\ldots ,\;s_{0}$, and $u^{*}=u_{1-p},u_{2-p},u_{3-p},\ldots ,\;u_{0}$ can easily be determined. The joint density of y given $s^{*}$ and $u^{*}$ can be calculated by conditional likelihood function is given by

(12) $$L_{y|s^{*},u^{*}}\left(\varphi ,\theta ,\delta ,\sigma^{2}\right)={\left(\frac{1}{\sqrt{2\pi }\sigma }\right)}^{n}\exp\begin{array}{c} \left\{-\frac{1}{2\sigma^{2}}\sum_{a=1}^{T}u_{a}^{2}(s^{*},u^{*},\varphi ,\theta ,\delta )\right\} \end{array}\;={\left(\frac{1}{\sqrt{2\pi }\sigma }\right)}^{n}\exp\left\{-\frac{1}{2\sigma^{2}}\kappa^{*}\left(\theta ,\varphi ,\delta \right)\right\}$$

where

$$\kappa^{*}\left(\theta ,\varphi ,\delta \right)=\sum_{a=1}^{T}u_{a}^{2}(s^{*},u^{*},\varphi ,\theta ,\delta )$$

The conditional log-likelihood function can be calculated by taking ln on both sides.

(13) $$\begin{array}{cc} l_{y|s^{*},u^{*}}\left(\varphi ,\theta ,\delta ,\sigma^{2}\right) & =\ln(L_{y|s^{*},u^{*}}\left(\varphi ,\theta ,\delta ,\sigma^{2}\right))\\ \; & =-\frac{n}{2}-\frac{n}{2}\ln\left(\sigma^{2}\right)-\frac{1}{2\sigma^{2}}\overset{T}{\underset{a=1}{\sum }}u_{a}^{2}(s^{*},u^{*},\varphi ,\theta ,\delta ) \end{array}$$

(14) $$\;\;\;\;=-\frac{n}{2}-\frac{n}{2}\ln\left(\sigma^{2}\right)--\frac{1}{2\sigma^{2}}\kappa^{*}\left(\theta ,\varphi ,\delta \right)$$

The values that maximize $l_{y|s^{*},u^{*}}\left(\varphi ,\theta ,\delta ,\sigma^{2}\right)$ and $L_{y|s^{*},u^{*}}\left(\varphi ,\theta ,\delta ,\sigma^{2}\right)$ are the values $\hat{\varphi },\hat{\theta },\hat{\delta }$ that minimize $\kappa^{*}\left(\theta ,\varphi ,\delta \right)=\sum_{a=1}^{T}u_{a}^{2}(s^{*},u^{*},\varphi ,\theta ,\delta )$ with ${\hat{\sigma }}^{2}=\frac{1}{n}\sum_{a=1}^{T}u_{a}^{2}(s^{*},u^{*},\hat{\varphi },\hat{\theta },\hat{\delta })=\frac{1}{n}\kappa^{*}\left(\hat{\theta },\hat{\varphi },\hat{\delta }\right)$.

## 3. Proposed Algorithm

**Figure 2:**
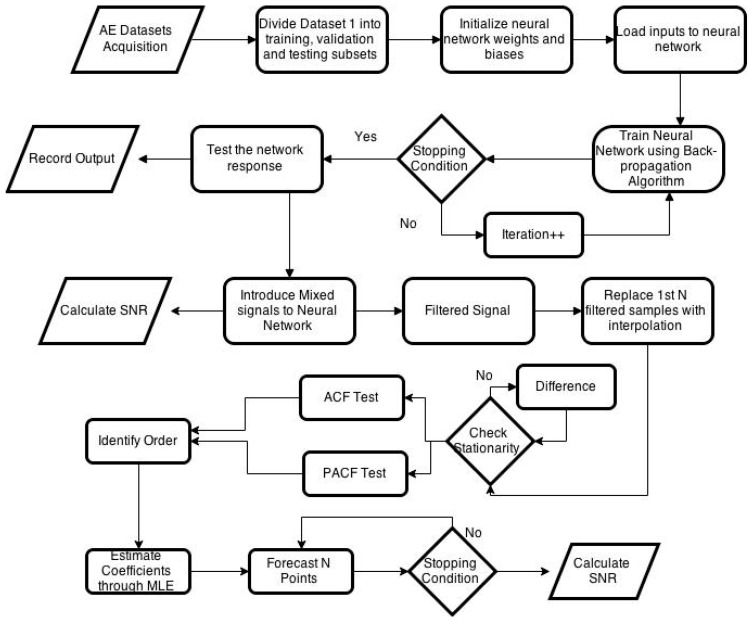
Flowchart of the proposed algorithm.

Airborne acoustic emission signals can be affected by a variety of background noise signals. However, in the proposed method noise from only four background machines are considered and are taken as noise. Figure 2 shows the flowchart of the algorithm. Acoustic signals from the machining process as well as from background machines are recorded separately. Statistical features such as mean, standard deviation, RMS, skewness, kurtosis, signal maximum, and minimum values are calculated. To reduce the background noise, a feedforward neural network is proposed to use as an adaptive filter. A machining signal could be filtered through traditional filters; however, the traditional filters would fail within the characteristic frequency range. The network is trained with individual acquired signal features using the procedure defined in Section 2. Afterward, background noise signals for one second is introduced to the machining signal. The noisy signal is then used to evaluate the performance of the neural network as an adaptive filter. The trained network works as an adaptive filter and removes noise samples from the signal. To reconstruct the filtered signal automatically, an ARMA-based algorithm is then developed.

**Figure 3:**
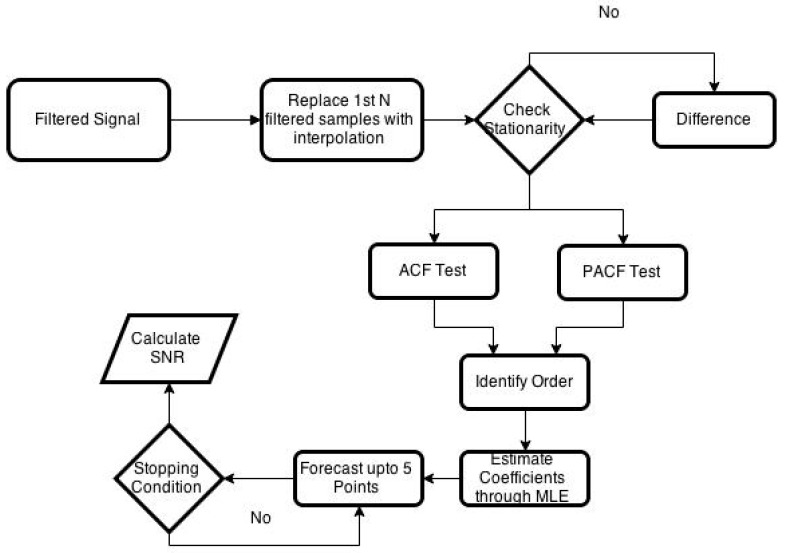
ARMA-based reconstruction algorithm flowchart.

The first step in the signal reconstruction algorithm is to identify the ARMA model order. To determine the order of the ARMA model, ACF and PACF tests are used for which at least 50 samples from the signals are needed. For the first 50 samples, the sample values of the machining signal that are filtered falsely by the algorithm are replaced by the values determined by the average of the two neighboring samples. After replacing the falsely filtered values, the ARMA model order is estimated using the first 50 samples. Once the model is identified, the ARMA model coefficients are found using MLE for the 50 samples. Once, the ARMA model is built, it is then used to forecast N sample points to reconstruct the filtered signal. The developed algorithm can forecast up to five sample points without moving the window further. To reconstruct more than five sample points, the algorithm first forecast five samples and then move the window to the next 50 samples and then check for several filtered samples. The process continues until the whole signal is reconstructed. After a complete signal is reconstructed, the reconstruction algorithm takes the average of the forecasted or reconstructed samples and update the false negatives values of the first 50 samples. Flowchart of the reconstruction algorithm is given in Figure 3.

## 4. Experimentation

**Figure 4:**
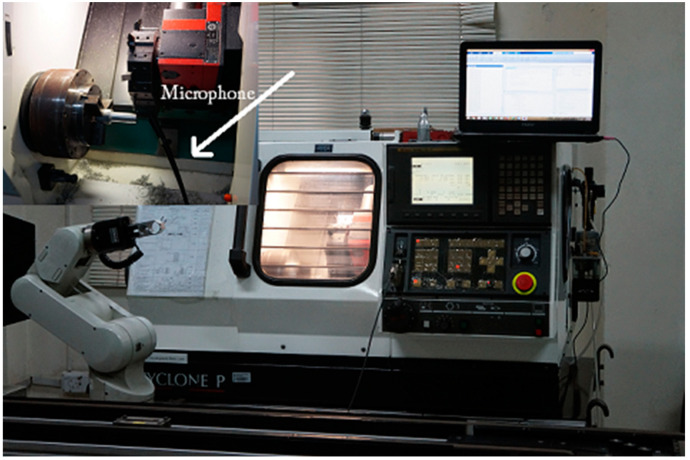
Experimental setup.

Experimentation is performed at Industrial Automation Lab of the college of EME, NUST (Pakistan). For a turning process, acoustic signals are acquired from a Denford Cyclone P CNC machine tool. Figure 4 shows the experimental setup.

The signals are acquired through an acoustic sensor at a rate sampling rate of 44.1 kHz to fulfill the Nyquist criteria. Each signal is recorded for 10 s. A total of 27 machining signals are recorded at different speeds and feed rates while depth of cut is kept constant. Calculated statistical features of the acquired data are tabulated in Table 1. The statistical properties of an acoustic signal such as first four moments, maximum value, minimum value, and maximum value of its root mean square (RMS) are taken as input.

Machining signals are recorded at different speeds and feed rates while depth of cut is kept constant. Calculated statistical features of the acquired data are tabulated in Table 1. The statistical properties of an acoustic signal such as first four moments, maximum value, minimum value, and maximum value of its root mean square (RMS) are taken as input.

Feature vectors to the neural network. The first and second moment of the signal are mean and standard deviation whereas the third and fourth moment about the mean are known as skewness and kurtosis [35]. The ${𝒾}^{th}$ standardized moment *ζi* of an acoustic signal s  is given as

(15) $$\zeta_{𝒾}=E\left[{\left(s-\bar{s}\right)}^{𝒾}\right]$$

and skewness of a signal is given by 𝒾=3 and can be defined as

(16) $$S_{k}=\frac{\zeta_{3}}{\sigma^{3}}$$

where *ζ* is the moment and is the standard deviation.

The signal skewness determines the signal symmetry about its mean value. Similarly, kurtosis of a signal is given by 𝒾=3  and can be defined as

(17) $$K_{x}=\frac{\zeta_{4}}{\sigma^{4}}$$

**Figure 5:**
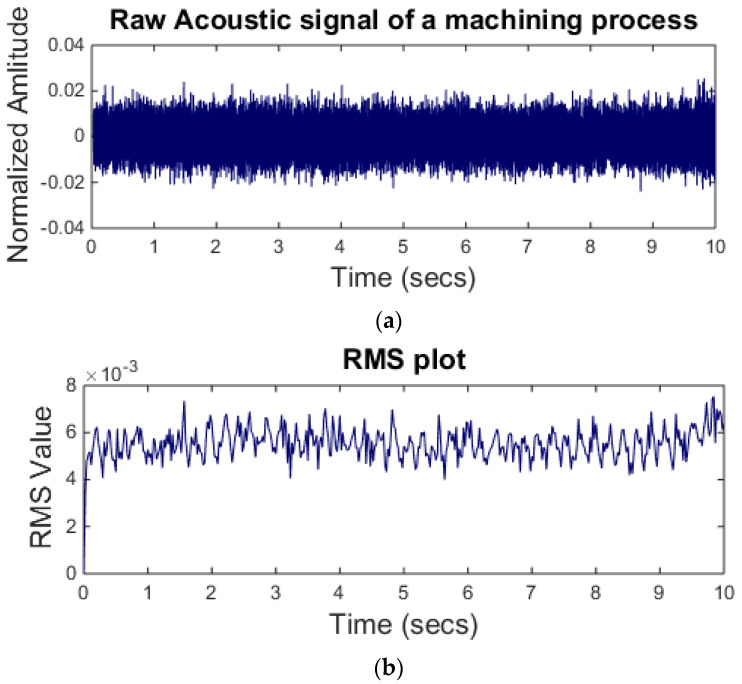
(**a**) Raw acoustic signal (above); (**b**) RMS level of the signal.

Kurtosis of a signal determines the sharpness of the peak of the signal. RMS of a machining signal varies with the increase in degradation of tool health. Hence, it is considered as a significant feature to monitor tool health [36,37]. RMS of a signal for a specific window length is defined as

(18) $$S_{rms}=\sqrt{\frac{s_{1}^{2}+s_{2}^{2}+\cdots +s_{nn}^{2}}{nn}}$$

where *s* is signal sample and nn is the total number of sample points in a window. Figure 5a shows a raw acoustic signal. The signal is acquired during a turning process at a feed rate of 200 mm/s and a constant depth of cut of 0.5 mm. To estimate the signal RMS in the time domain a window length of 1000 samples is selected. The RMS of the signal is shown in Figure 5b.

**Figure 6:**
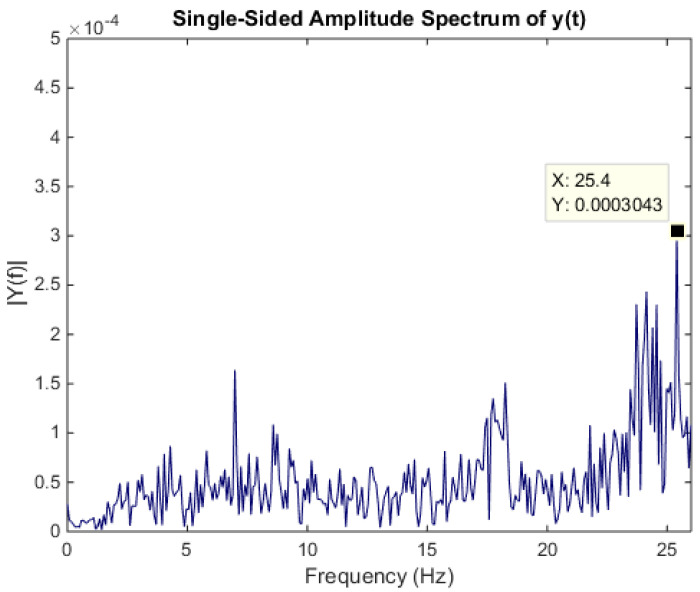
FFT plot of machining signal.

To validate the acquired signal from the microphone, the fast Fourier transform (FFT) of the machining signal is calculated. For the purpose, the signal is acquired at 1522 RPM which corresponds to 25.4 Hz in frequency domain. A clear peak at 25.4 Hz frequency can be seen in the Figure 6.

The machining signal can be affected by various types of environmental noise—for example, if an operator sneezes or a telephone rings in the background, etc. However, for this research, only parallel running machine noise is considered. Normally different machines operate in parallel on a shop floor. The acoustic signals of these parallel operating machines interfere with the acoustic signal of the machining process. These acoustic signals are a key source of the noise to the machining process acoustic signal. The acoustic signals from the parallel operating machines are considered as background noise. In this regard, four machines—a variable speed DC motor, a grinding machine, a three-axis CNC milling machine, and a four-axis mini milling machine—are selected and their signals are recorded. These machines are denoted as M1, M2, M3, and M4 while the CNC machine is denoted as MC. The distance between the CNC machine and the background machines is fixed; however, each machine has a specific distance from the CNC machine. The distance between the CNC machine and background machines are given in Table 2.

**Figure 7:**
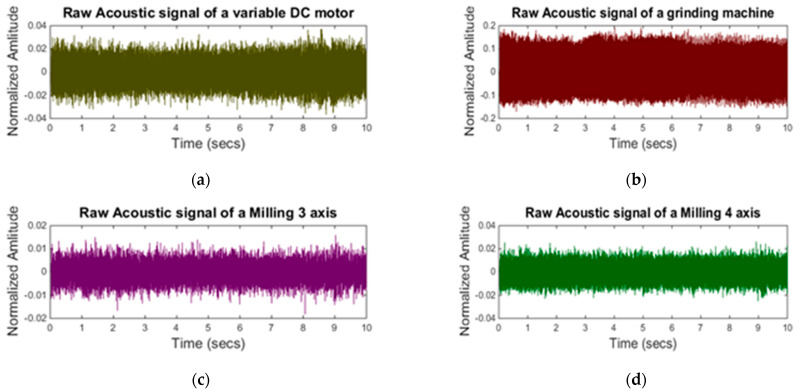
Raw acoustic signals of background noise: (**a**) variable DC motor; (**b**) grinding machine; (**c**) milling three–axis; (**d**) milling four-axis.

**Figure 8:**
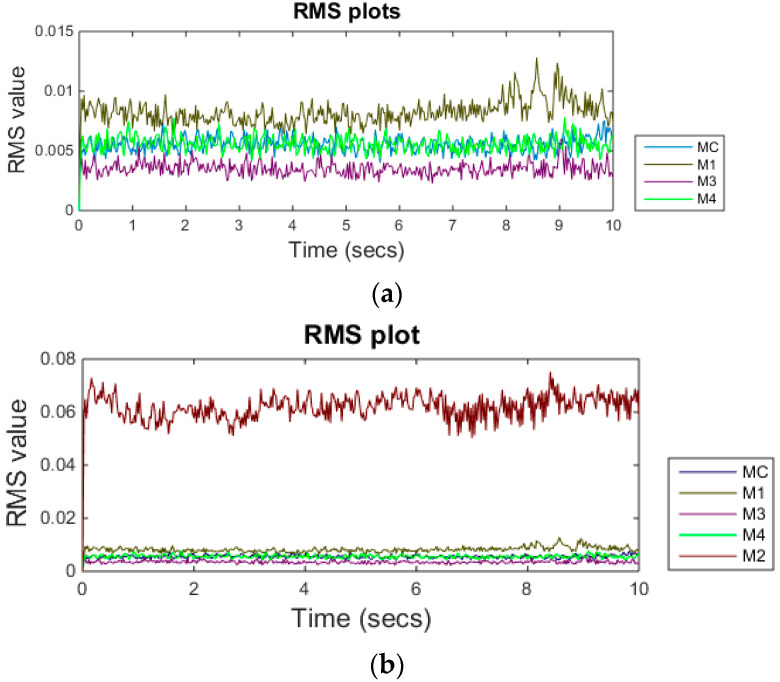
(**a**) RMS plot of machines excluding M2 (above); (**b**) RMS plot of all machines (below).

Figure 7 shows the acoustic signals of the selected background machines while Figure 8 shows the RMS level of the signals. It can be seen from Figure 8a that machine 4 signal has overlapping RMS level with machining signal RMS. As it is clear from Figure 8b that the grinding machine signal was quite dominant and its RMS level is much higher than the rest of the machine signals.

The neural network is trained with a single dataset A whereas the performance of the trained neural network is tested on two separate datasets namely dataset A and data B. Both datasets are prepared from the same (foreground and background) machines. For dataset A and B, machining signals and signals from each background machine having a length of 10 s are recorded. Dataset A consists of a total of 881 RMS samples. Out of 881 samples, 441 samples are from machining signal while 440 samples are taken from background machines signal (110 samples from each machine). A separate set of signals from the machining as well as background machines is also recorded and is termed as dataset B. This signal set is used to test the neural network performance. Similar to dataset A, the RMS level of the signals is calculated and taken as an important feature. Hence, dataset B consists of 2205 RMS samples with equal 441 samples from machining signals as well as from the background machine.

## 5. Results and Discussion

The architecture of the neural network consists of 4, 10, and 1 neurons in the input, hidden layer, and output layer respectively. Dataset A is further divided into three sub-datasets with 70% samples used for training purposes, 15% used for validation purpose, and remaining data used for testing. The neural network is trained until the minimum value of the validation error of 0.1124 is found. The performance of the neural network is assessed using dataset B. For machining signal, the neural network showed an accuracy of 82.3%. The confusion matrix of the back-propagation neural network for machining signal is given in Table 3. The table shows that when only machining signal is fed to trained neural network, it classifies 363 samples correctly. In the table, class 1 represents machining signal, whereas, class 0 represents the background noise or signals from other sources. The neural network showed an accuracy of 96.1% for M1 machine. Accuracy in the case of M2 is found to be maximum (100%) for grinding machine. This is due to the fact that the acoustic signal of M2 is quite distinguishable and dominant. Accuracy in the case of M4 is found to be 95.7%. The neural network showed the worst performance for M3 and shows an accuracy of only 5%. One of the possible reasons for the low accuracy of M3 is that the RMS level of M3 is overlapping with machining signal RMS as shown in Figure 8a.

**Figure 9:**
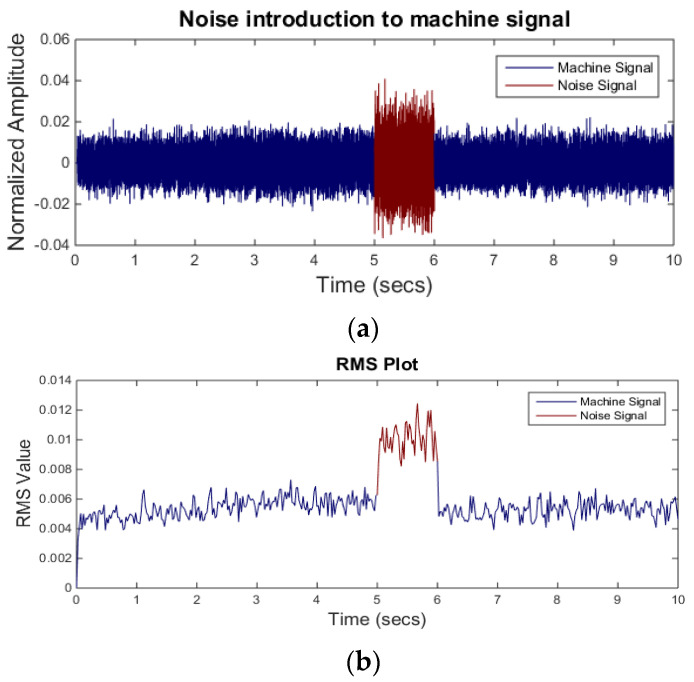
(**a**) Noise introduction (above) and (**b**) RMS level of the same signal (below).

Until now, the neural network is tested on limited number of signal samples. To demonstrate the accuracy of the neural network in real scenario, a separate dataset (dataset B) is used. For the dataset B, the background noise signal is mixed with the machining signal for one second. Different combinations of the background noise are used to validate the network performance in various scenarios. A machining signal mixed with the M4 noise is shown in Figure 9. In the figure, the machining signal mixed with the background noise is shown in red color in the 5–6 s windows. It can be seen that the RMS level of the signal between 5 and 6 s is higher than the rest of the signal.

**Figure 10:**
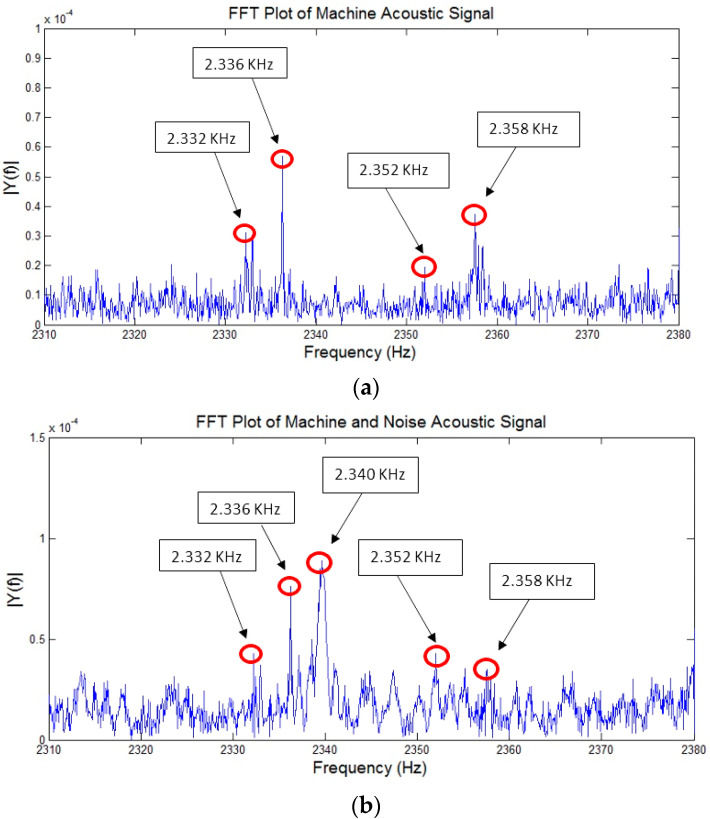
(**a**) FFT plot of an acoustic signal before noise addition (above); (**b**) FFT plot of an acoustic signal after noise addition (below).

FFT plot of the machining signal is shown in Figure 10a before adding the noise. Four dominant peaks of the machining signal at 2.332 kHz, 2.336 kHz, 2.352 kHz, and 2.358 kHz can be seen in the figure. Figure 10b shows the FFT of the machining signal after mixing with background noise. It can be seen from Figure 10b that an additional peak at 2.340 kHz is added to the plot. In addition to that it can also be observed that amplitudes of the dominant peaks are also affected by the noise. Hence, it validates the hypothesis that signal cannot be analyzed in the frequency domain as the background noise frequency may fall within the machining signal frequency of interest.

**Figure 11:**
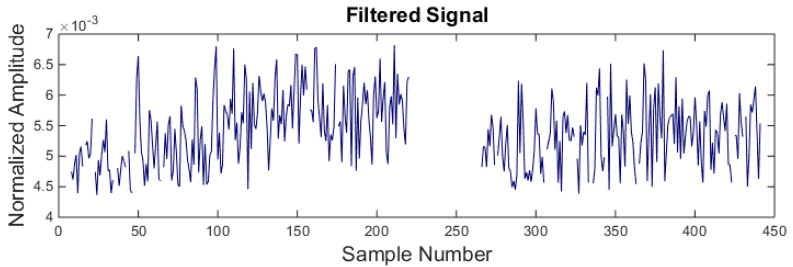
Signal filtration using back-propagation neural network.

The noisy signal is then passed through the back-propagation neural network. Figure 11 shows the filtered signal. It is observed that RMS values between 220 and 264 sample numbers which correspond to the noisy patch in the signal are filtered. However, some other values from the machining signal are also wrongly classified.

To reconstruct the filtered signal a time series model ARMA-based algorithm is developed. To estimate the order of the ARMA model, a minimum of 50 points is needed; therefore, to make the signal continuous or to reconstruct the filtered values for the first 50 samples, the filtered values are initially replaced by the average of the two neighbor samples. A variance test has been used to check the stationarity of the signal. The variance of the signal is calculated and recorded for the series and then the series is differenced until minimum variance is found which is equal to 1. Therefore, one difference in the series is taken. Table 4 shows the different values of variance calculated at a different order of difference. It can be seen that variance is minimum at D1.

**Figure 12:**
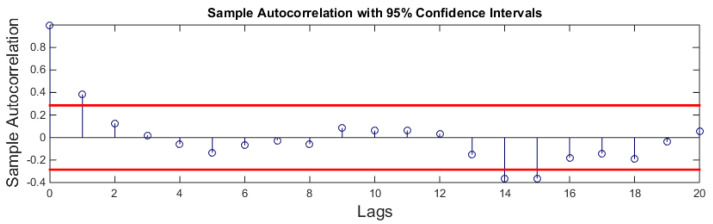
ACF plot.

ACF and PACF tests are used to calculate the order of MA and AR models respectively. Figure 12 shows the ACF plot of the signal. ACF is calculated for 20 lags only. It can be seen that only one peak at the lower lags is above the upper bound of the graph. This suggests that the MA model should be of order 1.

**Figure 13:**
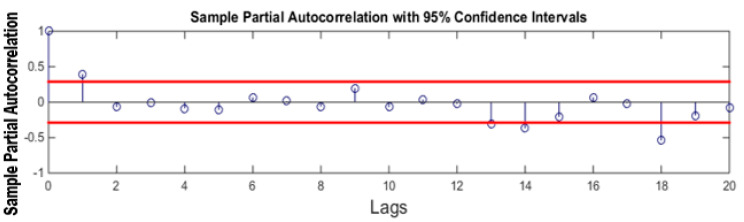
PACF Plot.

Figure 13 shows the PACF plot of the signal which is also calculated for 20 lags only. A peak of 0.4 is found at lag 1 which is suggesting the AR model of order 1. However, ARIMA models with *p* = 1,2,3,4 and *q* = 1,2,3,4 are also investigated and the best results are found are ARIMA (2,1,2).

The coefficient values for the selected model are found using maximum likelihood estimation. The values of the coefficients are tabulated in Table 5.

**Figure 14:**
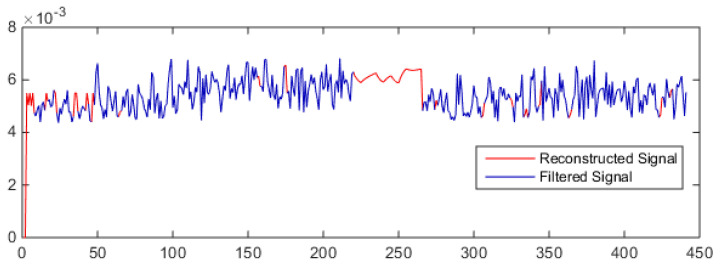
Reconstructed signal.

The estimated ARMA model is then used to reconstruct the filtered signal. A window of 50 samples is selected and then a maximum of five samples are forecasted to reconstruct the signal. Then the window is moved to the next 50 values of the signals and the same procedure is repeated until the complete signal is reconstructed. After that, filtered values for the first 50 samples which were replaced by the average of their neighbor samples, are now replaced by the average of the filtered signal. Figure 14 shows the reconstructed signal.

In the above figure, the machining signal is shown by a blue line while the filtered or reconstructed samples are shown by the red line. The neural network results are presented in Table 6. In the table, the first column represents the various combinations of machining and noise signals. For example, MC + M1 shows that the machine 1 signal is introduced to the machining signal. In order to validate the proposed algorithm performance, three indicators—namely, signal to noise ratio, coefficient of variation, and mean square error—are used. The signal to noise ratio (SNR) is the ratio between mean and standard deviation of a signal and it is given as

(19) $$\mathrm{SNR}=\frac{\bar{S}}{\sigma_{s}}=\frac{\frac{1}{N}\sum_{i=1}^{N}s_{i}}{\sqrt{\frac{1}{N}\sum_{i=1}^{N}\left(s_{i}-\bar{S}\right)}}$$

where $\bar{S}$ and $\sigma_{s}$ are the mean and standard deviation of the signal. Coefficient of variation (CV) is the reciprocal to SNR. In the Table 6, the fourth column SNR1 represents SNR of the mixed signal before filtering. The SNR2 column represents the SNR of the filtered and reconstructed signal. Similarly, the CV1 column represent coefficient of variation of the mixed signal whereas CV2 column represent the coefficient of variation of the filtered and reconstructed signal. From Table 6, it can be observed that the SNR of the machining signals before and after adding the noise are significantly improved. Similarly, the CV value of the machining signal reduces after filtering. Column 9 of the table shows the percentage increase in the SNR before and after filtering the noise. The last column of the table shows mean squared error between filtered signal and the original machining signal.

## 6. Conclusions

Airborne acoustic emission from a machining process provides vital information regarding the tool wear and can be used to develop a low-cost solution to monitor tool health. However, background noise is one of the major challenges in its implementation. The research aims to propose a solution to filter the noise generated by parallel running machines on the shop floor to overcome the implementation challenge of airborne acoustic emission in the lab environment. The characteristic frequency of tool condition for airborne acoustic emission lies within the first few kHz. Parallel running machining machines frequency can lie within or outside this range. Traditional band-pass, band-stop, or notch filters can be applied if noise frequency lies outside the machining frequency range; however, these filters would also filter the machining signal if the noise frequency lies within the machining frequency range. Therefore, different techniques of machine learning algorithms have been used as adaptive filters to classify machine and noise signals.

Different combinations of noise signals are mixed and introduced to machine signals to test the performance of the proposed solution in a real environment. To reconstruct the filtered signal, the ARMA-based algorithm is used. A series needs to be stationary to estimate the ARMA model. Proper order of differencing is selected using variance and autocorrelation function to avoid under and over differencing of the series. An under-differenced series would behave as a non-stationary series, while on the other hand, it is difficult to estimate over-differenced series coefficients. Order of moving average and autoregressive model is estimated to be around 1 using autocorrelation and partial autocorrelation function test, however, better results are found at ARIMA (2, 1, 2). ARMA-based signal reconstruction shows promising results with an average increase in SNR of 70.3% before and after signal reconstruction. Moreover, an average MSE of 1.3 × 10^−7^ is found between reconstructed and original machining signal. Finally, it can be concluded that the proposed technique can work best for the lab environment; however, the proposed technique can face a challenge to filter the noise having characteristics closer to the machining signal. Moreover, implementation of ARMA-based reconstruction algorithm in the dominant noisy environment would be a challenging problem.

## Figures and Tables

**Table 1:** Various features of acquired signals.

Test No.	Sample No.	Speed (RPM)	Feed Rate (mm/s)	Mean $\times 10^{-5}$	Standard Deviation	Max	Min	RMS Max	Kurtosis	Skew-Ness
1	1	1016	200	−1.74	0.0044	0.0191	−0.0152	0.1137	2.9318	0.0136
2	2	1016	200	−1.81	0.0046	0.0192	−0.0161	0.0063	2.9340	0.0195
3	3	1016	200	−1.70	0.0045	0.0194	−0.0151	0.0058	2.9543	0.0038
4	1	1016	400	−1.56	0.0052	0.0235	−0.0196	0.0091	3.1213	0.0011
5	2	1016	400	−1.63	0.0049	0.0561	−0.0212	0.0071	3.1899	−0.0258
6	3	1016	400	−1.54	0.0054	0.0274	−0.0213	0.0095	3.1457	0.0251
7	1	1016	600	−1.63	0.0055	0.0298	−0.0253	0.0108	3.3991	0.0611
8	2	1016	600	−1.85	0.0046	0.0202	−0.0161	0.0062	3.0040	0.0439
9	3	1016	600	−1.48	0.0068	0.0460	−0.0444	0.0201	4.3357	−0.0548
10	1	1522	200	−1.74	0.0055	0.0222	−0.0219	0.0079	2.9679	0.0087
11	2	1522	200	−1.62	0.0056	0.0255	−0.0194	0.0075	3.0014	0.0120
12	3	1522	200	−1.53	0.0060	0.0277	−0.0269	0.0107	3.1795	−0.0256
13	1	1522	400	−1.28	0.0061	0.0407	−0.0273	0.0119	3.2998	−0.0347
14	2	1522	400	−1.41	0.0055	0.0865	−0.0212	0.0109	4.0949	0.0081
15	3	1522	400	−1.62	0.0054	0.0233	−0.0182	0.0075	2.9845	0.0071
16	1	1522	600	−1.95	0.0062	0.0396	−0.0424	0.0158	3.9978	−0.0397
17	2	1522	600	−1.85	0.0056	0.0256	−0.0213	0.0088	3.0727	0.0117
18	3	1522	600	−1.61	0.0057	0.0347	−0.0233	0.0092	3.0663	0.0153
19	1	2000	200	−1.50	0.0057	0.0250	−0.0200	0.0083	2.9056	0.0158
20	2	2000	200	−1.68	0.0059	0.0279	−0.0227	0.0091	2.9688	−0.0105
21	3	2000	200	−1.58	0.0059	0.0292	−0.0283	0.0096	3.0455	0.0503
22	1	2000	400	−1.33	0.0055	0.0249	−0.0195	0.0083	2.9997	0.0364
23	2	2000	400	−1.72	0.0055	0.0244	−0.0199	0.0079	3.0126	0.0463
24	3	2000	400	−1.56	0.0057	0.0249	−0.0209	0.0078	3.0517	0.0340
25	1	2000	600	−1.64	0.0061	0.0287	−0.0223	0.0101	2.9768	0.0330
26	2	2000	600	−1.47	0.0063	0.0269	−0.0234	0.0089	2.9606	0.0501
27	3	2000	600	−1.51	0.0063	0.0318	−0.0289	0.0121	3.2031	0.0403

**Table 2:** Background machine distance measures.

Machine	Symbol	Approx. Distance
A variable speed DC motor	M1	6 m
Grinding machine	M2	3 m
Three-axis milling machine	M3	1 m
Four-axis mini-milling machine	M4	3 m

**Table 3:** Confusion matrix for machining signal.

**Predicted** **Class**	**Output Class**		
	Class	0	1
	0	0	72
	1	0	363

**Table 4:** Variance value at a different order of difference.

Order of Difference	Variance
Variance	$4.76\times 10^{-7}$
D1	$4.70\times 10^{-7}$
D2	$9.37\times 10^{-7}$
D3	$2.51\times 10^{-6}$
D4	$8.09\times 10^{-6}$

**Table 5:** Estimated coefficients values.

Model	Coefficient 1	Coefficient 2
Autoregressive model (∅)	−0.0186	0.6104
Moving Average model (θ)	−0.1894	−0.8105

**Table 6:** Results for real scenarios.

Noise	MEAN1$\times 10^{-2}$	STD1$\times 10^{-2}$	SNR1	CV1	MEAN2$\times 10^{-3}$	STD2$\times 10^{-4}$	SNR2	CV2	% Increase	MSE $\times 10^{-7}$
MC + M1	0.5812	0.1619	3.590	0.278	5.447	6.97	7.810	0.128	54.02	1.34
MC + M2	1.0439	1.5243	0.684	1.460	5.447	6.97	7.810	0.128	91.23	1.34
MC + M3	0.551	0.0903	6.098	0.163	5.478	7.32	7.486	0.133	18.53	1.68
MC + M4	0.5634	0.1161	4.851	0.206	5.478	7.29	7.513	0.133	35.43	1.72
MC + M1 + M2	1.0501	1.5425	0.680	1.468	5.447	6.97	7.810	0.128	91.28	1.34
MC + M1 + M3	0.588	0.1809	3.250	0.307	5.447	6.97	7.810	0.128	58.38	1.34
MC + M1 + M4	0.5959	0.2025	2.943	0.339	5.447	6.97	7.810	0.128	62.31	1.34
MC + M2 + M3	1.0456	1.5291	0.683	1.462	5.447	6.97	7.810	0.128	91.24	1.34
MC + M2 + M4	1.0473	1.5351	0.682	1.46	5.447	6.97	7.810	0.128	91.26	1.34
MC + M3 + M4	0.5707	0.1345	4.242	0.235	5.447	6.97	7.810	0.128	45.67	1.34
MC + M1 + M2 + M3	1.0519	1.5476	0.679	1.471	5.447	6.97	7.810	0.128	91.29	1.34
MC + M1 + M2 + M4	1.0535	1.5532	0.678	1.474	5.447	6.97	7.810	0.128	91.31	1.34
MC + M1 + M3 + M4	0.6018	0.2191	2.746	0.364	5.447	6.97	7.810	0.128	64.83	1.34
MC + M2 + M3 + M4	1.0491	1.5397	0.681	1.467	5.447	6.97	7.810	0.128	91.27	1.34
MC + All Noise	1.0553	1.5581	0.677	1.476	5.447	6.97	7.810	0.128	91.32	1.34

## Data Availability

Not applicable.
